# Implementation and adaptation of the Society of Thoracic Surgeons´ standardized nomenclature for congenital heart surgery in a Peruvian hospital

**DOI:** 10.47487/apcyccv.v6i3.489

**Published:** 2025-09-24

**Authors:** Marina Huamán Robles, Samuel Y Kim, Rachel Bernier, Ruht Villarroel Villa, Carlos Carcausto Huamaní, Arnaldo Munive Méndez, Rosina Ruiz Roque, María Isabel Picón Perla, Luis Vera Talledo, Tommy Prado Gómez, Arcelia Reyes Barriga, Corina Céspedes Solano, Víctor Justo Robles Velarde, Celia Mendoza Peltroche, Miriam Gaby Escate, Gloria Bernedo Gómez, José Luis Yovera, Rodrigo López Barreda, Juan Ibla

**Affiliations:** 1 Instituto Nacional Cardiovascular, Lima, Peru. Instituto Nacional Cardiovascular Lima Peru; 2 Boston Children´s Hospital, Boston, EEUU. Boston Children´s Hospital Boston EEUU; 3 Universidad Peruana de Ciencias Aplicadas (UPC), Lima, Peru Universidad Peruana de Ciencias Aplicadas Universidad Peruana de Ciencias Aplicadas (UPC) Lima Peru; 4 Pontificia Universidad Católica de Chile, Santiago, Chile Pontificia Universidad Católica de Chile Pontificia Universidad Católica de Chile Santiago Chile; 5 Ann & Robert H. Lurie Children's Hospital of Chicago, Chicago, EEUU Ann & Robert H. Lurie Children's Hospital of Chicago Chicago EEUU

**Keywords:** Database, Cardiac Surgical Procedures, Heart Defects, Congenital, Peru, Base de Datos, Procedimientos Quirúrgicos Cardíacos, Cardiopatías Congénitas, Perú

## Abstract

**Objective.:**

To describe the process of implementing a congenital heart surgery database at the National Cardiovascular Institute (INCOR) in Peru, adapted to the standardized nomenclature of the Society of Thoracic Surgeons, and to present clinical-surgical outcomes in terms of morbidity and mortality within the framework of comprehensive multidisciplinary care.

**Materials and Methods.:**

A prospective observational study was conducted in three phases: feasibility, database construction, and prospective data collection. Variables were adapted to the institutional context, and the process included a pilot test, strategic planning, multidisciplinary training, role assignment, monitoring, and periodic statistical analysis.

**Results.:**

A total of 500 consecutive patients were registered between May 2022 and July 2023. Septal defects were the most common surgical indication. The most frequent complications included cardiac dysfunction and arrhythmias. In-hospital mortality was 7.6%. The database incorporated preoperative nutritional and dental assessments. Although 27.2% of the forms were fully completed, the remainder showed limitations such as missing data and the need for additional verification of information across modules.

**Conclusion.:**

The implementation of a congenital heart surgery registry in Peru is feasible even in a resource-limited setting. This system allows the identification of critical areas for improving care and optimizing clinical outcomes, establishing a foundation for future quality improvement initiatives.

## RESUMEN

**Objetivo.:**

Describir el proceso de implementación de una base de datos para cirugía cardíaca en pacientes con cardiopatías congénitas en el Instituto Nacional Cardiovascular (INCOR) en Perú, adaptada a la nomenclatura estandarizada de la Society of Thoracic Surgeons y presentar los resultados clínico-quirúrgicos en morbilidad y mortalidad, en el marco de una atención multidisciplinaria integral.

**Materiales y métodos.:**

Estudio observacional, prospectivo desarrollado en tres fases: viabilidad, construcción de la base de datos y recopilación prospectiva. Se adaptaron variables al contexto institucional; el proceso incluyó prueba piloto, planificación estratégica, capacitación multidisciplinaria, asignación de roles, monitoreo y análisis estadístico periódico.

**Resultados.:**

Se registraron 500 pacientes consecutivos entre mayo de 2022 y julio de 2023. Los defectos septales fueron la principal indicación quirúrgica; las complicaciones más frecuentes incluyeron disfunción cardíaca y arritmias. La mortalidad hospitalaria fue del 7,6 %. La base de datos permitió integrar la evaluación nutricional y odontológica preoperatoria. Si bien el 27,2% de los formularios en la base de datos estaba completo, el resto presentó limitaciones como datos faltantes y la necesidad de verificación adicional de la información entre módulos.

**Conclusión.:**

Es factible implementar un registro de cirugía cardíaca congénita en Perú con recursos limitados. Este sistema permite identificar áreas críticas para mejorar la atención y optimizar los resultados clínicos, estableciendo una base para futuras iniciativas de mejora en la calidad asistencial.

## Introduction

Congenital heart disease (CHD) is a significant cause of childhood morbidity and mortality. Its global prevalence is estimated at 9.4 per 1000 live births, with a sustained increase in recent decades due to improved prenatal and neonatal diagnosis. ^1^ Despite advances in surgical treatment, CHD continues to impose a substantial health burden, particularly in low- and middle-income countries, where postoperative mortality rates may exceed 10%. ^2^

In North America, Europe, and Oceania, consolidated multicentre registries have made a substantial contribution to improving care in congenital cardiac surgery. Notable examples include the Society of Thoracic Surgeons Congenital Heart Surgery Database (STS-CHSD) ^3^, the European Congenital Heart Surgeons Association Database (ECHSA) ^4^, and the Australia and New Zealand Congenital Outcomes Registry for Surgery (ANZCORS). ^5^ These registries apply standardised nomenclatures, such as that of the Society of Thoracic Surgeons-European Association for Cardio-Thoracic Surgery (STS-EACTS), which enable longitudinal patient follow-up, rigorous analysis of clinically relevant outcomes, and the implementation of improvements in clinical practice. In addition, they foster active participation of multidisciplinary teams, facilitate outcome comparisons, support risk assessment, and establish quality standards tailored to their respective regions.

In 2005, the Congenital Cardiac Anesthesia Society (CCAS) developed specific recommendations for anaesthesia in CHD, which were subsequently integrated into the STS database. This initiative evolved in 2013 into a multi-institutional project that compiles standardised data from more than 100 centres across the USA, Canada, and Japan, enabling the systematic recording of clinical and surgical information to improve practice and outcomes. ^6^

In contrast, in South America, most centres lack structured systems and uniform methodologies for the clinical-surgical registration of patients with CHD. The absence of standardised criteria for variable definitions, data collection, and follow-up scheduling results in considerable heterogeneity across institutions, limiting the feasibility of consistent multicentre analyses and hindering regional collaboration to improve outcomes. ^7^

In Peru, the available information on congenital cardiac surgery remains limited, particularly regarding perioperative care characteristics related to anaesthetic management, surgical technique, perfusion, and postoperative care. ^8^ In this context, the establishment of an institutional clinical-surgical database represents a significant step forward.

The aim of this study is to describe the implementation process of a cardiac surgery database for patients with CHD in Peru, adapted from the STS model, designed to capture standardised information on anaesthesia, surgical procedures, and clinical outcomes, including postoperative complications and follow-up results.

## Materials and methods

### Study design

An observational, descriptive, prospective study conducted at the National Cardiovascular Institute (INCOR)-EsSalud between May 2022 and July 2023.

This initiative was developed in collaboration with the Division of Cardiac Anesthesia at Boston Children’s Hospital (BCH), which provided technical sponsorship and administrative support for the development and implementation of the registry, to systematise clinical information and optimise outcomes in this population.

To establish a system for data recording and storage, we used the standardised variable structure of the STS congenital heart surgery database as reference. ^9^ Version 3.41, updated on May 5, 2020, was translated into Spanish and validated by the authors to facilitate its use in the local context, thereby enabling more efficient operational implementation.

**Figure 1:**
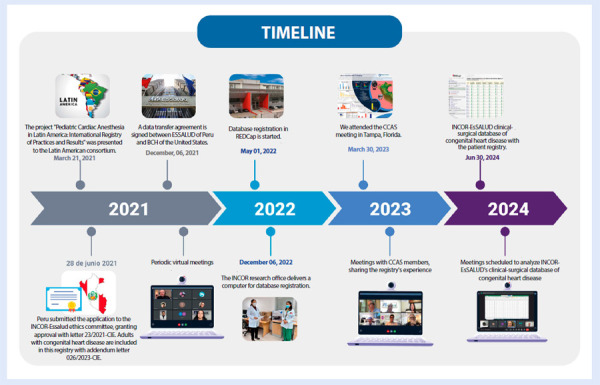
Implementation of the clinical-surgical database for congenital heart disease at INCOR-EsSalud.

Source: INCOR-EsSalud Congenital Heart Disease Registry.

The implementation process was established through a collaborative effort in three phases (Figure 1):

INCOR: National Cardiovascular Institute. EsSalud: Social Health Insurance. BCH: Boston Children’s Hospital. CCAS: Congenital Cardiac Anesthesia Society. REDCap: Research Electronic Data Capture.

Feasibility phase: technical meetings were held with a multidisciplinary team comprising a designated representative from paediatric cardiology, cardiac surgery, cardiovascular anaesthesia, perfusion, paediatric dentistry, and nutrition. Through clinical and operational consensus, the original STS registry modules were reviewed, key variables adaptable to the Peruvian context were identified, and additional variables deemed relevant based on local experience were incorporated. This adaptation process was essential to ensure that the database accurately reflected the clinical, structural, and operational particularities of the local setting. The final version of the registry was internally validated, ensuring its operational feasibility and prospective applicability, consistent with other international experiences of implementing and standardising clinical registries in congenital cardiac surgery. ^10^

Construction of the REDCap-EsSalud BCH database: the database was structured using the Research Electronic Data Capture (REDCap) software ^11^, integrating a final version of the variables organised into nine modules that facilitated comprehensive case analysis and provided a complete overview of the care process. This platform enables the systematic and secure recording of all patient-related information, covering the preoperative, intraoperative, and postoperative phases, as well as follow-up through the first year.

Prospective data collection phase: after the initial pilot test, prospective and consecutive data collection was initiated by the research team and clinical collaborators. Patients were identified from the daily list of scheduled surgeries provided by the Division of Cardiovascular Anaesthesia and the Surgical Centre, and were entered into REDCap following data coding and anonymisation.

From the outset of the registry’s implementation, investigators and clinical collaborators entered information once patient care was completed. Each specialist recorded only the data corresponding to their area within the module assigned to their specialty in the REDCap platform.

To optimise the registry system, a pilot test was conducted during the first month of implementation, including 45 patients. This stage allowed the identification of errors in data entry and the establishment of clearer criteria for recording surgical reinterventions during the same hospitalisation, both those requiring cardiopulmonary bypass and those that did not (e.g., haemostasis revision, surgical debridement, or diaphragmatic plication). This initial organisation facilitated improved capture and analysis of these events within the database.

**Figure 2:**
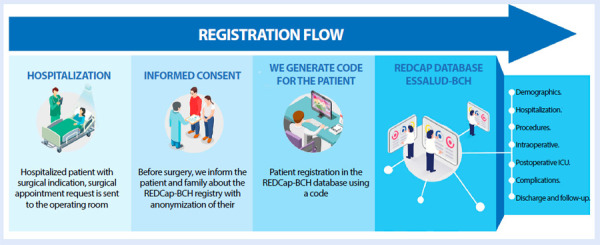
Patient registration flow for congenital heart disease.

Patient follow-up was conducted at 30 days, and at 3, 6, and 12 months after surgery, through two approaches: in-person outpatient visits and telephone calls made by the same team of investigators and designated collaborators, who entered the corresponding information directly into the follow-up modules of the REDCap platform (Figure 2).

EsSalud: Social Health Insurance. BCH: Boston Children’s Hospital. REDCap: Research Electronic Data Capture.

The primary source of clinical information was the institutional medical record, in which diagnoses were established based on specialised clinical evaluation and cardiovascular imaging studies. Surgical indication was determined by a multidisciplinary team. Our institution uses the International Classification of Diseases, 10th Revision (ICD-10), as the standard system for diagnostic coding ^12^, although this coding is not applied directly in the REDCap platform. In the absence of a genetics laboratory, the identification of chromosomal, genetic, or clinical syndromic anomalies was based on clinical evaluation by the care team or on documentation recorded at hospital admission.

### Study population

We included 500 consecutive patients with a confirmed diagnosis of CHD, ranging from newborns to adults, without age restriction, who underwent surgical intervention with or without the use of cardiopulmonary bypass between May 2022 and July 2023. As all patients operated on during this period were included, no sampling was performed; instead, the entire available population was analysed. Only patients who provided informed consent were considered; for those under 18 years of age, consent was obtained from parents or legal guardians.

### Variables

Divided into nine modules, with main variables (Table 1). Age was categorised by group: neonates (0-29 days), infants (30 days to 2 years), preschool and school-age children (2-10 years), adolescents (10-18 years), and adults (>18 years).

**Table 1:** Structure of modules in the INCOR-EsSalud congenital heart disease registry.

N.°	Module	Main content
1	Demographic	Place of origin, perinatal history, syndromic or genetic data
2	Hospitalisation	Laboratory tests, imaging studies and cardiac catheterisation, nutritional and dental assessment
3	Diagnosis	Diagnostic classification and coding adopted from the IPCCC
4	Procedure	Each procedure coded according to the IPCCC
5	Intraoperative	Surgical times, cardiopulmonary bypass, use of blood products
6	Anaesthesia	nvasive/non-invasive monitoring, drugs administered, anaesthetic adverse events
7	Postoperative	ICU management, haemodynamic support, postoperative procedures
8	Complications	Postoperative adverse events, reinterventions
9	Discharge and follow-up	Discharge information, readmissions, follow-up at 30 days, 3, 6, and 12 months

Source: INCOR-EsSalud congenital heart disease registry.

IPCCC: International Pediatric and Congenital Cardiac Code. ICU: Intensive Care Unit.

The variables with specific measures recorded preoperatively included the presence of dental caries by age group in the dental assessment, and nutritional status by anthropometric evaluation (weight/height/body mass index [BMI]), classified as follows: underweight (below the 5th percentile on growth charts); at risk of underweight (children with a fall of two or more percentiles on growth charts); normal weight (children and adolescents between the 5th and 85th percentiles on appropriate growth charts); at risk of obesity (children trending towards higher percentiles; for adults, BMI: 25-29.9 kg/m²); and obesity (children and adolescents above the 95th percentile on growth charts, and adults with BMI ≥30 kg/m²). ^13^

The diagnosis and procedure module collects clinical information using the standardised STS classification, with coding based on the International Pediatric and Congenital Cardiac Code (IPCCC). ^10^ This system allows for the structured recording of both anatomical diagnoses of CHD and surgical procedures performed, facilitating international data comparison and uniform outcome analysis. In our implementation, the codes were translated into Spanish and adapted to the local context, while preserving equivalence with the original database. 

### Procedures and interventions

The creation of the database combined the standardised structure of the STS registry with operational implementation in the REDCap platform, following a process organised in several stages. Initially, the local adaptation of STS variables was carried out through technical meetings of a multidisciplinary team, which selected and translated the variables relevant to the institutional context. The final version was organised into nine modules addressing the different phases of care: preoperative, intraoperative, postoperative, and follow-up.

Subsequently, digital forms were developed in REDCap, including structured fields and basic entry logic, which enabled the start of prospective data collection. The research team and clinical collaborators manually entered data after each surgical procedure using the REDCap web-based platform.

During this process, internal training sessions, pilot testing, and periodic reviews of data entry were conducted. The platform facilitated centralisation of information and its export for subsequent analyses. By the end of 2022, INCOR had joined the international REDCap consortium, ensuring the continuity of the clinical-surgical registry system for patients with CHD and its institutional use.

### Ethical aspects

The project was submitted to the Research Office and the Ethics Committee of INCOR, receiving approval through letter No. 23/2021-CEI on June 28, 2021. Subsequently, administrative procedures were completed at the central level within EsSalud, culminating in the signing of a data transfer agreement between EsSalud and Boston Children’s Hospital (BCH), which was accepted on December 21, 2021. Initially, approval covered only paediatric patients with CHD, but it was later extended through addendum No. 26/2023-CEI to include adult patients with this condition. Written informed consent was obtained from all participants: directly in the case of adults, and from parents or legal guardians for paediatric patients (under 18 years). Each patient underwent a coding and anonymisation process, in accordance with ethical principles of data protection and current institutional regulations, ensuring confidentiality and privacy of personal information at all times.

### Statistical analysis

The database was developed and managed using REDCap, which enabled the systematic collection of clinical information through structured forms.

Quality control was carried out manually by exporting the data to Microsoft® Excel® (version 2503) to correct frequent errors, such as the use of incorrect characters (“O” instead of “0”), inconsistent decimal notation (commas instead of points), empty or miscoded fields, and, in particular, date-entry errors, which represented one of the most common challenges.

After data cleaning was completed, the dataset was exported to Stata/MP 14.0 for descriptive statistical analysis. Categorical variables were summarised as absolute and relative frequencies (percentages). Numerical variables were described using measures of central tendency and dispersion, according to their distribution, although categorical data predominated in this section. Results were presented in tables and figures to facilitate interpretation.

## Results

A total of 500 consecutively registered patients were included between May 2022 and July 2023, ranging in age from 1 day to 55 years.

In evaluating the quality of the REDCap database, 500 records were analysed. Of these, 27.2% (n=136) were complete, whereas 12.0% (n=60) were empty. Additionally, 28.0% (n=140) required verification, and 6.2% (n=31) corresponded to patients with multiple admissions, that is, two or three hospitalisations in different periods. Moreover, 26.6% (n=133) of patients had heterogeneous records across modules; for example, complete information in some forms (such as surgery or perfusion), but empty or pending verification in others (such as follow-up or complications). Missing entries in certain modules necessitated a retrospective review of medical records to complete the data.

**Figure 3:**
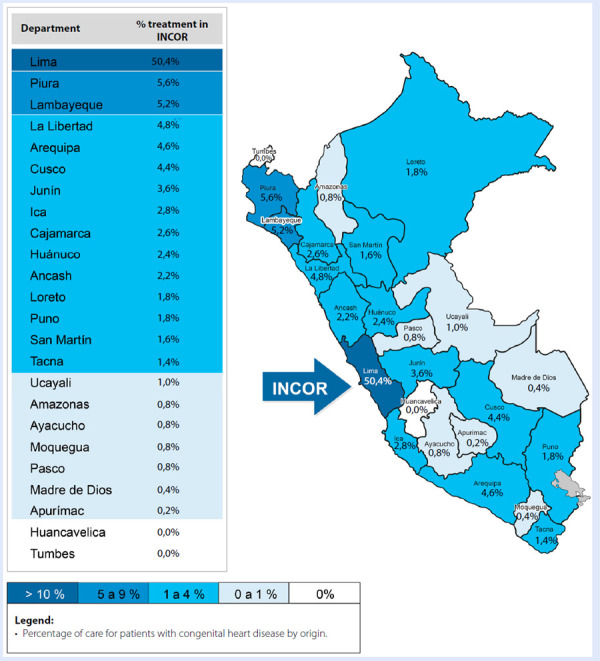
Geographical origin of patients treated at INCOR.

Source: INCOR-EsSalud Congenital Heart Disease Registry.

Regarding patient origin, Lima accounted for the largest proportion: 50.4% (n=252), followed by Piura with 5.6% (n=28), Lambayeque with 5.2% (n=26), and La Libertad with 4.8% (n=24). By contrast, regions such as Apurímac, Madre de Dios, and Moquegua contributed very few patients, each representing less than 0.4% (Figure 3).

INCOR: National Cardiovascular Institute. EsSalud: Social Health Insurance.

General characteristics of the study population are presented in Table 2. In addition, non-cardiac congenital anatomical anomalies were recorded in 10.6% (n=53), chromosomal or genetic anomalies in 19.4% (n=97), and syndromes in 22.2% (n=111). Of the 186 patients with dental caries, 10.8% (n=20) required general anaesthesia for dental clearance prior to surgical procedures.

**Table 2:** Clinical characteristics of patients undergoing surgery for congenital heart disease at INCOR, 2022-2023.

Variables	n (%)
Sex	
Female	242 (48.4%)
Male	258 (51.6%)
Prenatal diagnosis	
Yes	47 (9.4%)
No	453 (91.6%)
Haemoglobin levels (WHO classification)	
Normal	278 (55.6%)
Mild anaemia	151 (30.2%)
Moderate anaemia	15 (3.0%)
Severe anaemia	0 (0.0%)
Polycythaemia	56 (11.2%)
Dental caries	
Neonates	0 (0.0%)
Infants	9 (4.8%)
Preschool and school-age	108 (58.1%)
Adolescents	57 (30.6%)
Adults	12 (6.0%)
Nutritional status by anthropometry	
Neonates	At risk of underweight: 8 (16.0%) - Underweight: 11 (22.0%) - Normal weight: 27 (54.0%) - At risk of obesity: 4 (8.0%) - Obesity: 0 (0.0%)
Infants	At risk of underweight: 47 (21.7%) - Underweight: 125 (57.6%) - Normal weight: 41 (18.9%) - At risk of obesity: 4 (1.8%) - Obesity: 0 (0.0%)
Preschool and school-age	At risk of underweight: 26 (22.6%) - Underweight: 18 (15.7%) - Normal weight: 50 (43.5%) - At risk of obesity: 19 (16.5%) - Obesity: 2 (1.7%)
Adolescents	At risk of underweight: 12 (13.6%) - Underweight: 4 (4.5%) - Normal weight: 66 (75.0%) - At risk of obesity: 6 (6.8%) - Obesity: 0 (0.0%)
Adults	At risk of underweight: 0 (0.0%) - Underweight: 0 (0.0%) - Normal weight: 25 (83.3%) - At risk of obesity: 0 (0.0%) - Obesity: 5 (16.7%)

Source: INCOR-EsSalud Congenital Heart Disease Registry.

NYHA: New York Heart Association. INCOR: Instituto Nacional Cardiovascular. WHO: World Health Organization.

Among the surgical procedures performed, the most frequent were closure of septal defects (atrial septal defect [ASD], ventricular septal defect [VSD], atrioventricular [AV] canal, truncus arteriosus) in 42.5% (n=294). These were followed by procedures for right-sided lesions (tetralogy of Fallot, pulmonary valve disease, Ebstein anomaly, and tricuspid valve disease) in 14.7% (n=104); palliative procedures (Blalock-Taussig shunt, bidirectional/unidirectional Glenn, pulmonary artery banding, systemic-to-pulmonary shunts) in 12.1% (n=84); and interventions on thoracic arteries and veins (coarctation of the aorta, hypoplasia of the aortic arch, interrupted arch, and patent ductus arteriosus) in 9.0% (n=67). Less frequent procedures included left-sided lesions (aortic valve disease, mitral valve disease, and hypoplastic left heart syndrome) in 7.4% (n=50); pulmonary venous anomalies in 5.7% (n=35); single ventricle (Fontan procedures) in 3.1% (n=19); transposition of the great arteries in 1.8% (n=11); and double-outlet right ventricle in 1.8% (n=11). Mechanical circulatory support (Extracorporeal membrane oxygenation [ECMO], intra-aortic balloon pump, or ventricular assist device) was used in 1.6% (n=10), and two heart transplants were performed (0.3%, n=2).

Intraoperative and postoperative complications are shown in Table 3, while Table 4 presents hospital mortality, which was 7.6%.

**Table 3:** Anaesthesia-related adverse events and postoperative complications.

Variables	n (%)
Anaesthesia-related adverse events	
No events reported	389 (77.8%)
Vascular access	58 (11.6%)
Arrhythmia at the end of aortic cross-clamping	13 (2.6%)
Cardiac arrest - not related to anaesthesia care	6 (1.2%)
Cardiac arrest related to anaesthesia care	4 (0.8%)
Hypercyanotic episode (Tet spell)	4 (0.8%)
Complications during patient transfer	4 (0.8%)
Difficult intubation/reintubation	3 (0.6%)
Bronchospasm	3 (0.6%)
Postoperative complications	
No complications reported	207 (21.8%)
Cardiac dysfunction resulting in low cardiac output	136 (14.3%)
Arrhythmia requiring pacemaker	62 (6.5%)
Postoperative/procedural respiratory failure requiring mechanical ventilation >7 days	52 (5.5%)
Pulmonary hypertension requiring treatment	49 (5.2%)
Arrhythmia requiring medical treatment	41 (4.3%)
Sepsis	39 (4.1%)
Postoperative/procedural respiratory failure requiring reintubation	30 (3.2%)

Source: INCOR-EsSalud Congenital Heart Disease Registry.

**Table 4:** In-hospital mortality by age group.

Age group	Total n (%)	Alive n (%)	Deceased n (%)
Neonates (0-29 days)	50 (10.0%)	39 (78.0%)	11 (22.0%)
Infants (30 days-2 years)	192 (38.4%)	173 (90.1%)	19 (9.9%)
Preschool/School-age (2-10 years)	140 (28.0%)	134 (95.7%)	6 (4.3%)
Adolescents (10-18 years)	88 (17.6%)	86 (97.7%)	2 (2.3%)
Adults (>18 years)	30 (6.0%)	30 (100%)	0 (0.0%)
Total	500 (100%)	461 (92.4%) *	38 (7.6%)

Source: INCOR-EsSalud Congenital Heart Disease Registry.

In-hospital mortality at discharge. (*One patient was not operated on.)

## Discusión

The most relevant finding of this study was the feasibility of implementing a clinical-surgical database for congenital cardiac surgery in a national referral centre in Peru, which enabled the systematisation of information from 500 patients. Hospital mortality was 7.6% and reached 10% at 1 year, figures higher than those reported in consolidated international registries. ^14^ Critical deficiencies in comprehensive care were also identified: only 9.4% of cases were diagnosed prenatally, infants showed a high prevalence of underweight (57.6%) and nutritional risk (21.7%), with mild anaemia in 30.2%, and 81.4% of children had dental caries. These findings underscore the importance of adapting registries to local needs by incorporating nutritional and dental variables, which are not typically included in international databases.

The implementation of specialised databases is essential for the effective management of clinical data in congenital cardiac surgery, as it enables systematic integration of information, rigorous analysis of clinical outcomes, and improved decision-making in health care. Moreover, it promotes the standardisation of practices and contributes to the continuous improvement of quality of care ^15^. In the Peruvian context, the creation of a database inspired by international models such as the STS, but adapted to local particularities, represents a pioneering initiative with transformative potential for patient care.

The initial experience highlighted limitations such as incomplete records and pending data verification across modules. These challenges are common in early stages of implementation, particularly in resource-limited settings, and were also observed in consolidated databases such as the STS-CHSD and the ECHSA Database, which required continuous training, internal audits, and strict data validation to ensure quality. ^3^^,^^15^

In our study, only 9.4% of CHD cases were diagnosed prenatally, a figure considerably lower than the 30-60% reported in high-income countries, reflecting the limited availability of fetal echocardiography in prenatal care programmes and the absence of systematic screening. ^16^^,^^17^ Regarding nutritional status, infants showed the highest risk indicators: 21.7% had risk of underweight and 57.6% were underweight, with a prevalence of mild anaemia of 30.2%. These findings highlight the importance of comprehensive nutritional evaluation during preoperative planning. ^18^ International literature has reported even higher figures, with malnutrition ranging from 65% to 85% and iron-deficiency anaemia in up to 55% of paediatric patients with CHD. ^19^ Anaemia, particularly when associated with heart failure or pulmonary hypertension, is linked to an increased risk of malnutrition, consistent with the vulnerability observed in our study.

The inclusion of preoperative dental assessment revealed a high prevalence of dental caries, particularly among preschool and school-age children, with 81.4% (in primary or mixed dentition). This finding is consistent with the Global Burden of Disease Study 2017 ^20^, which estimates that 60-90% of school-age children have caries. Several studies have shown that children with CHD have a higher prevalence of caries compared with the general population, attributed to factors such as diets rich in simple sugars, fear of dental treatment, and the low prioritisation of oral health within comprehensive clinical care. ^21^

In our institution, perioperative records are documented in multiple forms belonging to different services, which fragments the information and complicates cross-sectional analysis of the clinical-surgical process. In addition, the exclusive reliance on the ICD-10 ^12^ limits the accuracy of coding for CHD, as it does not adequately capture its anatomical or surgical complexity. This limitation highlights the need to adopt more specific and standardised coding systems, such as the International Pediatric and Congenital Cardiac Code (IPCCC) ^10^, as recommended by leading international scientific societies.

At the national level, it should be noted that this study was carried out exclusively at INCOR-EsSalud, a level III-E national referral centre specialised in cardiovascular diseases. Consequently, the data reflect only the insured population treated in this institution, which limits the generalisability of the findings to the broader Peruvian health system, characterised by fragmentation and unequal coverage. Furthermore, access to specialised care at INCOR-EsSalud is geographically concentrated in Lima, which accounts for 50.4% of the total coverage, highlighting inequities in the distribution of EsSalud insurance across the country’s regions. ^22^

Despite the benefits of implementing a clinical database, relevant limitations remain. The lack of an institutional culture oriented towards systematic data recording, also evidenced in our study ^23^, is closely related to the workload of health personnel and the scarcity of human resources trained in database management and statistical analysis. This situation is compounded by limited familiarity with technological tools, restricting their effective use in clinical research. These barriers are particularly critical in resource-limited institutions, where the sustainability of a robust registry system requires investment in training, technological infrastructure, and collaborative work among stakeholders.

In conclusion, the implementation of a specialised database for congenital cardiac surgery in the Peruvian context represents a milestone in strengthening quality of care. The adaptation of international models, such as the STS, to local realities has enabled the structured, multidisciplinary integration of essential clinical and surgical information. This initiative has identified critical areas directly influencing outcomes, such as prenatal diagnosis, nutritional status, and oral health, thereby contributing to more timely and patient-centred care.

Beyond optimising clinical management, this database is projected as a strategic tool for research, quality monitoring, staff training, and benchmarking against international standards. Its consolidation not only fosters standardisation and continuity of care, but also lays the foundation for the creation of a national congenital heart disease registry, essential to reduce inequities, guide health policies, and raise the standard of surgical care for patients with congenital heart disease in Peru.
